# Gut Barrier Dysfunction and Bacterial Lipopolysaccharides in Colorectal Cancer

**DOI:** 10.1007/s11605-023-05654-4

**Published:** 2023-03-27

**Authors:** Qiang Li, Viktor von Ehrlich-Treuenstätt, Josefine Schardey, Ulrich Wirth, Petra Zimmermann, Joachim Andrassy, Alexandr V. Bazhin, Jens Werner, Florian Kühn

**Affiliations:** 1 Department of General, Visceral, and Transplant Surgery, Ludwig-Maximilians-University Munich, Marchioninistr. 15, 81377 Munich, Germany; 2 German Cancer Consortium (DKTK), Partner Site Munich, 81377 Munich, Germany; 3 Bavarian Cancer Research Center (BZKF), Partner Site Munich, 81377 Munich, Germany

**Keywords:** Colorectal cancer (CRC), Gut barrier, Lipopolysaccharides (LPS), Tight junctions (TJs), Metastatic disease

## Abstract

**Background:**

Inflammation is known to be an essential driver of various types of cancer. An increasing number of studies have suggested that the occurrence and development of colorectal cancer (CRC) are linked to the inflammatory microenvironment of the intestine. This assumption is further supported by the fact that patients with inflammatory bowel disease (IBD) are more likely to develop CRC. Multiple studies in mice and humans have shown that preoperative systemic inflammatory response is predictive of cancer recurrence after potentially curative resection. Lipopolysaccharides (LPS) are membrane surface markers of gram-negative bacteria, which induce gut barrier dysfunction and inflammation and might be significantly involved in the occurrence and development of CRC.

**Methods:**

A selective literature search was conducted in Medline and PubMed, using the terms “Colorectal Cancer”, “Gut Barrier”, “Lipopolysaccharides”, and “Inflammation”.

**Results:**

Disruption of intestinal homeostasis, including gut barrier dysfunction, is linked to increased LPS levels and is a critical factor for chronic inflammation. LPS can activate the diverse nuclear factor-κB (NF-κB) pathway via Toll-like receptors 4 (TLR4) to promote the inflammatory response, which aggravates gut barrier dysfunction and encourages CRC development. An intact gut barrier prevents antigens and bacteria from crossing the intestinal endothelial layer and entering circulation. In contrast, a damaged gut barrier triggers inflammatory responses and increases susceptibility to CRC. Thus, targeting LPS and the gut barrier might be a promising novel therapeutic approach for additional treatment of CRC.

**Conclusion:**

Gut barrier dysfuction and bacterial LPS seem to play an important role in the pathogenesis and disease progression of colorectal cancer and therefore require further investigation.

## Introduction

Colorectal cancer (CRC) is the third most frequent cause of cancer mortality worldwide, with a five-year survival rate of approximately 60%.^1^ Twenty percent of patients with CRC have metastatic disease when diagnosed, and 25% of diagnosed patients will develop metastasis in the further course of the disease.^1^ The development of CRC might be due to a combined effect of inflammation and immune regulation.^2–4^

It is well-known that patients suffering from inflammatory bowel disease (IBD) are at an increased risk of developing CRC.^5–7^ Thus, as a driver of gut inflammation, gut barrier dysfunction plays a crucial role in the inflammatory mechanism of CRC pathogenesis. The integrity of the gut barrier is maintained mainly by the structure of tight junctions (TJs) and adherens junctions (AJs) of the mucosal layer.^8,9^ TJs are composed of proteins such as claudin, occludin, and zonula occludens-1 (ZO-1), and AJs contain cadherins, α-catenin, β-catenin, and afadin.^10^ Lipopolysaccharides (LPS) are proinflammatory mediators from different types of gram-negative bacteria and are essential to their outer cell walls. They can change the integrity of the intestinal barrier by dysregulating the TJ proteins,^11,12^ leading to a gut barrier injury through an inflammatory response.^13–15^ The impaired intestinal barrier integrity permits bacteria-derived molecules and other antigens to cross the gut barrier to maintain this intestinal inflammation.^8,9,16^ LPS can enter the circulatory system and trigger inflammatory-immune responses after bacterial release in pathological conditions.^17^ The level of LPS in blood has previously been linked to systematic inflammation and various types of cancer.^18–20^ LPS activates inflammatory activity through a series of pathways. Within the intestine, it can activate the NF-κB pathway by TLR4 receptors to aid the inflammatory response.^21,22^ This major inflammatory pathway not only aggravates gut barrier dysfunction but might also contribute to the onset and development of CRC.^23^ In addition, LPS seems to affect different steps of CRC metastasis, like cell adhesion to the extracellular matrix (ECM), cell detachment due to ECM degradation, and cell invasion.^24^

Therapies targeting LPS have the potential to inhibit metastasis and improve the prognosis of CRC in vitro and in vivo.^25^ This article reviews the current literature regarding the role of LPS, the gut barrier, and therapies targeting LPS in CRC.

## The Role of Gut Barrier in CRC

### Gut Barrier Dysfunction Increases Susceptibility to CRC

The gut is lined with a barrier that acts as a physical and functional barrier, protecting against harmful agents, such as bacteria and toxins. Under physiological conditions with an intact gut barrier integrity, the tolerance and immune response to foreign antigens are balanced, and inflammation in the intestinal tissue is suppressed.^2^ However, when the gut barrier is damaged, it triggers the diffusion of small molecules and bacteria into the host systemic circulation and causes the expansion of inflammation and immunological disturbance.^3,4,26^ Data from mouse models shows that TJs and AJs are essential structures for maintaining the integrity of the intestinal barrier while their damage induces inflammation.^27,28^

CRC tumorigenesis is linked to an inflammatory microenvironment.^23^ The damaged intestinal barrier exposes intestinal stem cells to genotoxic compounds or environmental mutagens, which can significantly promote intestinal inflammation and increase the risk of cancer.^5,26^ In IBD patients, the incidence of intestinal barrier dysfunction is increasingly contributing to a chronic-inflammatory state which is a substantial risk factor for CRC.^6,29^ Substantial cellular damage to the intestinal barrier can not only be induced through intestinal inflammation. However, intestinal inflammation can further aggravate it and will mutually reinforce the effect of susceptibility to colorectal cancer.^27,28^ Therefore, damage to the intestinal barrier and intestinal inflammation appear to play a significant role in the genesis of CRC. Protecting the gut barrier has been proven effective in inhibiting CRC progression.^30^

### The Mechanism of Gut Barrier Disruption Increasing CRC Tumorigenesis

**Fig. 1:**
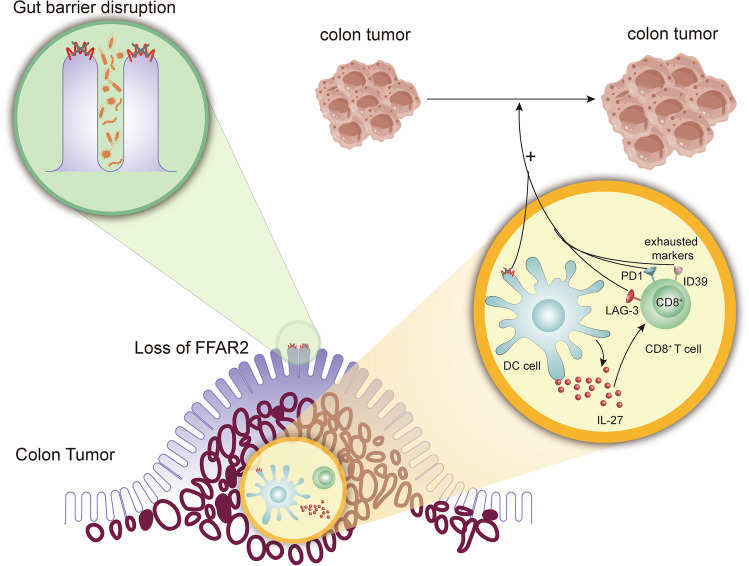
Role of the gut barrier in CRC. The gut barrier disruption is caused by increasing colon tumor-associated bacteria, which is regulated by the loss of FFAR2 in the intestinal epithelial cells. The gut barrier increases the susceptibility to colon cancer in the FFAR2-related pathway. In gut barrier disruption, the loss of FFAR2 on DCs overacts the DCs to increase the expression of IL-27, which is related to CD8 + T cell exhaustion

The primary cellular mechanism through which the gut barrier disruption is formed is the free fatty acid receptor 2 (FFAR2)-related pathway. FFAR2 widely exists in intestinal epithelial cells^31^ and maintains gut homeostasis and the integrity of the gut barrier.^32,33^ Previous works show that an FFAR2 deficiency increases susceptibility to CRC by threatening the integrity of the gut barrier.^33,34^ Its pathway plays a vital role in immune cell function, such as in dendritic cells (DC), participating in innate and adaptive immune responses.^35^ When the gut barrier gets disrupted by a deficiency of FFAR2, DCs overact, alter their phenotypes, and increase the expression of interleukin (IL)-27. The level of IL-27 significantly correlates with the exhaustion marker of CD8 + T cells.^33^ Overacting DCs and exhausted CD8 + T cells allow tumor growth.^33,36^ Therefore, gut barrier disruption is a prerequisite for FFAR2-induced CRC tumorigenesis and tumor growth (Fig. 1).

### Modulating the Gut Barrier Through Cancer-Related Genes Promotes CRC

Cancer inhibitory or procarcinogenic pathways also regulate CRC occurrence by influencing the intestinal barrier.^37,38^ For example, N-myc proto-oncogene protein (N-myc) downstream-regulated gene 2 (NDRG2) was found to regulate the structure of AJs.^27^ Furthermore, the loss of NDRG2 downregulated the expression of E-cadherin in mouse models. The decrease in e-cadherin expression destroys the structure of AJs, again promoting gut barrier dysfunction, eventually resulting in spontaneous colitis and possibly resulting in colitis-associated tumor occurrence and development.^27,37,39^

Another membrane receptor called tumor necrosis factor receptor 2 (TNFR2), associated with several different tumor entities, including CRC, promotes gut barrier disruption by deregulating the TJ proteins.^28^ Mechanistically, TNFR2 was found to upregulate the expression of the long isoform of myosin light chain kinase (MLCK) to impair TJs and disrupt the gut barrier. TNFR2 modulated gut barrier dysfunction-induced colitis, which is associated with CRC.^28,38,40^

## The Role of LPS in Stimulating Gut Barrier Dysfunction

**Fig. 2:**
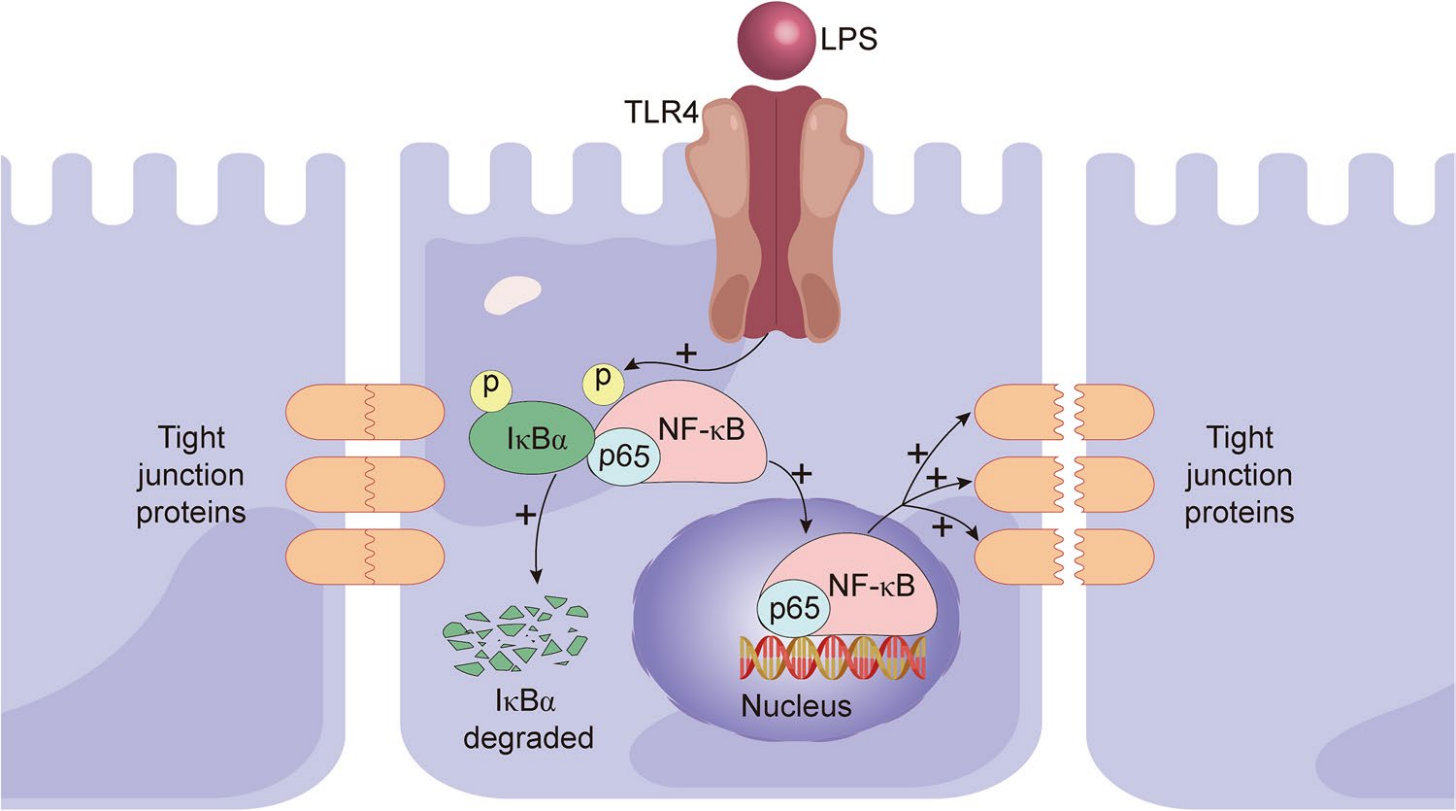
LPS stimulates gut barrier dysfunction by destroying tight junctions via the TLR4/NF-κB pathway. LPS-activated TRL4 promotes the phosphorylation of IκBa and p65, which results in p65 translocating to the nucleus and IκBa being degraded. The activated TLR4/NF-κB pathway eventually decreases the expression of TJ proteins and impairs the gut barrier

The integrity of the gut barrier is mainly maintained by TJs and AJs.^27,28^ Changes in TJs and AJs or the incompleteness of the mucosal layer will damage the intestinal barrier and increase intestinal permeability.^27,28^ In vitro and in vivo assays proved that LPS could induce inflammation and reduce intestinal epithelial occludin and claudin-1, essential components of TJs, to destroy the gut barrier.^12,13^ Regarding mechanism, LPS modulated gut barrier dysfunction by the TLR4/NF-κB inflammatory signaling pathway, during which the LPS-activated TRL4 played an integral part in the phosphorylation of IκBa and p65 in the cytoplasm. P65 translocates to the nucleus, while IκBa degrades, disrupting TJs.^13^ It has been shown that LPS from gram-negative bacteria, such as *S. marcescens* and *E. coli*, can control the gut barrier by changing TJ proteins.^11,12^ In conclusion, these examples show that intestinal LPS has the ability to disrupt the gut barrier (Fig. 2).

## The Role of LPS in the Carcinogenesis and Metastasis of CRC

### LPS-Related Signaling Pathways of CRC Metastasis

Bacterial LPS is a harmful agent that can contribute to the development of colorectal cancer when gut barrier dysfunction occurs. LPS-activated TLR4-related inflammatory signaling pathways play a crucial role in human cancer invasion and metastasis, affecting aspects such as the low survival rate of CRC.^41–43^ LPS is the primary activator of TLR4 in several cancer types, such as pancreatic, liver, and colorectal cancer. According to the analysis of 116 CRC patients, high expression of TLR-4 was associated with a high rate of metastasis and therefore related to poor prognosis.^44^ In vivo and in vitro assays also have proven that LPS promoted CRC cell adhesion and metastasis by mediating the TLR4 inflammatory signaling pathway.^45,46^

LPS-induced inflammation increases the possibility of metastasis in cancer.^47^ NF-κB has been considered an essential participant in the inflammatory response.^48^ LPS-induced intestinal epithelial cell inflammation increased the expression of adhesion molecules via the NF-κB pathway in vitro.^49^ LPS increased metastasis ability through various NF-κB-related pathways. For instance, the LPS-increased expression of HK3 by the NF-κB/Snail/HK3 signaling pathway promoted CRC metastasis in vitro and in vivo.^50^ LPS also promoted the invasion and metastasis of colon cancer cells by activating the NF-kB signaling pathway through the SDF-1a/CXCR4 axis in vitro and in vivo.^51^ Thus, clinically, CRC patients with high CXCR4 expression were more likely to have liver metastasis and a poor prognosis.^52,53^

### LPS Regulates Carcinogenesis and Metastasis in CRC

Current literature shows that LPS can induce or contribute to carcinogenesis, tumor progression, invasion, and metastasis in several types of cancer, including CRC.^42,54–56^ Due to changes in the gut flora and gut barrier, damage levels of LPS were increased in the intestine and portal venous blood of CRC patients compared to healthy people,^51^ which suggests that LPS has an effect on carcinogenesis in CRC. Especially in CRC metastasis, LPS could induce different steps, such as cell adhesion to the ECM, detachment due to ECM degradation, and invasion.^24^ Moreover, LPS in the tumor microenvironment (TME) could induce EMT.^57^ During EMT, epithelial cells acquired the phenotype of mesenchymal cells and fibroblasts and reduced their level of cell-to-cell adhesion, further inducing CRC progression.^58^ In vitro assays have proven that LPS stimulated colon cancer cells to express adhesion molecules.^59^ In addition, LPS increases liver metastasis of colon cancer cells in vivo.^45^

## Therapies Targeting LPS

Recent publications in the field investigate the clinical use of targeting LPS and its molecular pathways. As LPS shows a high abundance in primary CRC tissues,^25^ Song et al. designed an LPS-target fusion protein-coding sequence, which expressed an LPS trap protein after being loaded into a nanoparticle system in CRC tissue.^25^ Of note, LPS trap proteins prevented CRC liver metastasis in vivo.^25^ The tumor growth of LPS trap protein was inhibited even more pronounced after combining it with anti-PD-L1 therapy.^25^ Also, the LPS trap protein prolonged the survival time of CRC-bearing mice. Therefore, targeting LPS-induced CRC metastasis might improve the prognosis of CRC in vivo.

In addition, there are also commonly used drugs that show an effect by directly or indirectly targeting LPS. Metformin, considered a widely used drug for type 2 diabetes patients, plays a role in preventing systemic inflammation response.^60^ Studies showed that metformin inhibited the NF-κB phosphorylation activated by LPS to prevent CRC metastasis.^50^ Other commonly used medicines could attenuate LPS-stimulated CRC development.^61^ Morphine is a commonly used medicine for advanced cancer patients to relieve pain when it maintains a specific blood concentration.^61^ However, 0.1 or 1 μM of morphine can potentially alleviate the stimulation of LPS to tumor cells.^61^ Morphine can deregulate the expression of ICAM-1, VCAM-1, and E-selectin on HUVECs to decrease the expression of adhesion molecules on HUVECs under the stimulation of LPS in vitro.^61^ This finding suggested that morphine could attenuate the colon cancer metastasis stimulated by LPS. Aspirin, famous for its function as an antipyretic analgesic, also inhibits cancers such as breast, lung, ovarian, stomach, and colorectal.^62–64^ It attenuates the metastasis ability and decreases the EMT phenotype induced by LPS in vitro.^65^

Variations of fruits, food, and plant extracts also have an antitumor effect against LPS and CRC.^66–69^ Previous studies have found that apple polysaccharides could decrease cancer risk to a certain point, including CRC.^70^ The modified apple polysaccharides (MAP) functioned to inhibit metastasis of CRC against the cell migration process.^66^ In terms of mechanism, MAP prevented CRC metastasis and invasion by inhibiting the expression of COX2, iNOS, and MMP, which were all related to cell proliferation, cell apoptosis, angiogenesis, and cell invasiveness, in the LPS-activated NF-kB pathway.^66^ Besides, the γ-oryzanol-rich (OR) fraction hexane soluble fraction (HSF) in red and purple rice inhibited the invasion ability in vitro.^71,72^ In terms of mechanism, OR-rich HSF reduced cell adhesion to ECM and relieved cell detachment due to reduced expression of MMP-2, finally preventing cancer cell invasion.^67^ In addition, decursin, a pyranocoumarin from *Angelica gigas*, attenuated LPS-induced inflammation through TRL4 and JNK signaling, suggesting that decusin might be a kind of medicine that could be treated for LPS-caused disease such as CRC.^69^

The anti-inflammatory gut brush border enzyme intestinal alkaline phosphatase (IAP) is an essential regulator of intestinal homeostasis; it detoxifies LPS, stabilizes the natural intestinal flora, and regulates the barrier function of the intestine.^73–79^ In addition, IAP has been shown to play an essential regulatory role in glucose and lipid metabolism and counteract metabolic syndrome development.^80,81^ Reduced IAP activity has been measured in chronic diseases such as diabetes mellitus, liver cirrhosis, cardiovascular disease, and older age. In mouse models, oral supplementation with IAP resulted in a significantly longer lifespan and markedly reduced frailty.^82^ The anti-inflammatory function of IAP on innate immunity in humans has already been tested in clinical trials demonstrating its efficacy, for example, in blocking endotoxemia in septic patients.^83^ Based on its functions, IAP could hold an additional therapeutic option as a supplement in cancer patients in order to prevent or reduce a gut permeability-related or LPS-induced metastatic spread.

## Conclusion and Perspective

In conclusion, gut barrier dysfunction and bacterial LPS appear to affect CRC development through an increased inflammatory immune response. Changes in the gut barrier can trigger tumor-related inflammation and immune responses while intestinal inflammation promotes gut barrier disruption and increases susceptibility to CRC. Further research is needed to understand the exact mechanisms by which gut barrier dysfunction and bacterial LPS contribute to the development of CRC. Targeting LPS and gut barrier dysfunction by inhibiting intestinal inflammatory pathways, whose activation is a prerequisite for gut barrier injury, could be a new additional therapeutic strategy for CRC treatment.
